# Metatranscriptomics for Understanding the Microbiome in Food and Nutrition Science

**DOI:** 10.3390/metabo15030185

**Published:** 2025-03-10

**Authors:** Christina F. Butowski, Yash Dixit, Marlon M. Reis, Chunlong Mu

**Affiliations:** Smart Foods and Bioproducts, AgResearch, Te Ohu Rangahau Kai, Palmerston North 4474, New Zealand; nina.butowski@agresearch.co.nz (C.F.B.); marlon.m.reis@agresearch.co.nz (M.M.R.)

**Keywords:** metatranscriptomics, food fermentation, flavour formation, dietary fibres, nutrition, microbiome

## Abstract

Microbiome science has greatly expanded our understanding of the diverse composition and function of gut microorganisms over the past decades. With its rich microbial composition, the microbiome hosts numerous functionalities essential for metabolizing food ingredients and nutrients, resulting in the production of active metabolites that affect food fermentation or gut health. Most of these processes are mediated by microbial enzymes such as carbohydrate-active enzymes and amino acid metabolism enzymes. Metatranscriptomics enables the capture of active transcripts within the microbiome, providing invaluable functional insights into metabolic activities. Given the inter-kingdom complexity of the microbiome, metatranscriptomics could further elucidate the activities of fungi, archaea, and bacteriophages in the microbial ecosystem. Despite its potential, the application of metatranscriptomics in food and nutrition sciences remains limited but is growing. This review highlights the latest advances in food science (e.g., flavour formation and food enzymology) and nutrition science (e.g., dietary fibres, proteins, minerals, and probiotics), emphasizing the integration of metatranscriptomics with other technologies to address key research questions. Ultimately, metatranscriptomics represents a powerful tool for uncovering the microbiome activity, particularly in relation to active metabolic processes.

## 1. Introduction

The microbiome is a collection of microorganisms, genetic materials, and their biochemical activities. Recent advances in microbial culture and next-generation sequencing techniques have greatly broadened our understanding of microbial functionality. From a biochemical standpoint, the microbiome can produce a wide array of enzymes that metabolize various substrates, including carbohydrates/fibres [1], protein/amino acids [2,3], minerals [4], xenobiotics [5], and drugs [6]. These metabolic outputs constitute functional metabolites with varying properties. For instance, acetate and lactate serve as important flavour molecules during food fermentation [7], while they are also of great relevance in regulating gut and even neurological health [8,9].

Community profiling-based techniques have presented high-throughput, high-resolution opportunities in this context. The widespread application of 16S ribosomal RNA sequencing has contributed greatly to our understanding of the microbial structure [10,11]. To gain functional views of the microbiome, metagenomics, metatranscriptomics, and metaproteomics have been developed [12,13,14]. Among these technological innovations, metagenomics studies the collection of microbial DNA in a community, offering insights into taxonomic composition and functional potential, while metatranscriptomics focuses on the collection of messenger RNA (mRNA) from a microbial community, which can offer specific insights into microbial transcriptional activities. Proteomics specifically assesses proteins expressed by a microbial community, providing views on microbial enzymes and proteins involved in active processes. Depending on the research hypothesis, these approaches can be used either singularly or in combination as integrative omics to provide deeper views on microbial functionalities. Recently, mRNA-based metatranscriptomics sequencing has emerged as a powerful and unbiased approach for studying diverse microbial populations, as well as active functional processes involved.

In the field of human microbiome, metatranscriptomics has been employed to understand the role of gut microbiome in inflammatory bowel diseases [15,16,17], obesity [18], familial type 1 diabetes [19], metabolic associated fatty liver disease [20], and oral microbiome in periodontal disease [21], including supragingival plaque [22]. These findings have expanded our understanding of how gut microbiome contributes to pathogenesis and offer evidence to develop microbiome-based interventions.

However, in the field of food and nutrition science, the application of metatranscriptomics is relatively at the infancy stage. Current evidence has uncovered the role of the microbiome in food fermentation [23] and the regulation of dietary nutrients, food ingredients, food chemicals, and emulsifiers, as discussed below. To understand how to integrate metatranscriptomics into food and nutrition science, this review will synthesize available findings to dive into the biological mechanisms related to food fermentation and the dietary regulation of the gut microbiome.

## 2. Metatranscriptomics Basics

**Figure 1:**
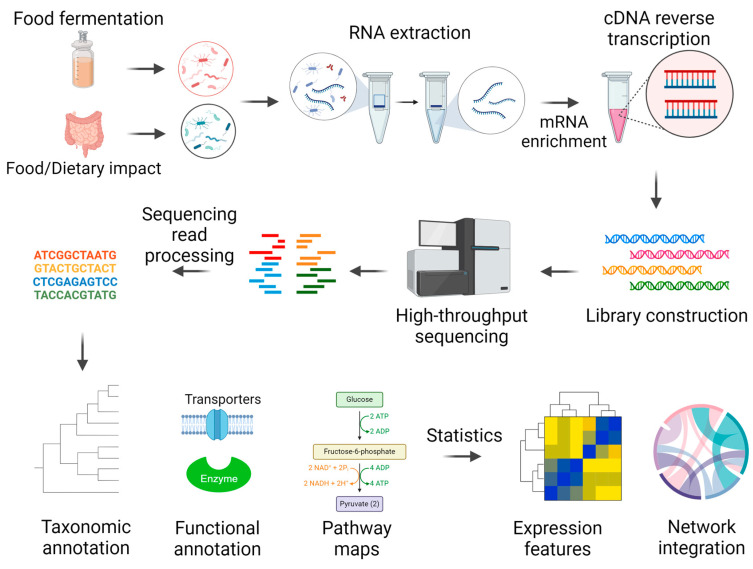
Metatranscriptomics pipeline.

Metatranscriptomics studies the collection of messenger RNA (mRNA) transcripts from microbial communities, which can infer specific insights into microbial transcriptional activities. Metatranscriptomics processes include microbiome RNA extraction, mRNA enrichment, reverse transcription to cDNA, high-throughput sequencing, and bioinformatics analyses (Figure 1). This bioinformatics pipeline requires adequate computing capacity to process sequencing reads, taxonomic classification, functional annotation, and statistical analysis for discriminant features, pathways, or modules.

## 3. Metatranscriptomics Methodology

### 3.1. RNA Extraction and mRNA Enrichment

Extraction of RNA from microbiome samples is the first critical step in metatranscriptomics processing. A major technical challenge is the low presence of mRNA in total cellular RNA (1–5%), with the left being ribosomal RNA (rRNA) and transfer RNA (tRNA). Different from mRNA in eukaryotes, prokaryotic mRNA does not contain a poly-A tail, making it impossible to benefit from oligod(T)-based RNA manipulation. The presence of rRNA in sequencing data increases sequencing costs and reduces coverages. Different methods to remove rRNA, such as subtractive hybridization (MICROBExpress Bacterial mRNA Enrichment kit, Thermo Fisher Scientific, Waltham, MA, USA) and exonuclease digestion (mRNA-ONLY Prokaryotic mRNA Isolation kit, Epicentre Biotechnologies, Madison, WI, USA), have been evaluated, where subtractive hybridization was found to be more sufficient in yielding quantitative data [24]. In this sense, other probe hybridisation-based kits have also been developed, such as riboPOOLs (siTOOLs Biotech, Planegg/Martinsried, Germany) and RiboMinus (Thermo Fisher Scientific, Waltham, MA, USA), with riboPOOLs being most successful in reducing the rRNA content [25]. Currently, a variety of rRNA removal kits are available commercially for lab users.

Nevertheless, another great challenge is RNA contamination from host cells. This is particularly important for a specimen where there is a high rate of host cell turnover, such as luminal or mucosal samples from the small intestine. Currently, the hybridization capture technology is available to remove mammalian RNA (MICROBEnrich™ Kit, Thermo Fisher Scientific, Waltham, MA, USA). In combination with an rRNA removal kit, these technical innovations make it possible to increase sequencing output and prokaryotic coverage. Genomic DNA may be a potential contamination during extraction, but it can be easily removed with DNase I-based methods.

### 3.2. Library Construction and Sequencing

After RNA extraction and mRNA enrichment, the fragmented RNA will be reverse-transcribed into double-stranded complementary DNA (cDNA) for sequencing. Since microbial mRNAs are mostly non-polyadenylated, random priming, such as random hexamers, is commonly employed in first-strand cDNA synthesis. RNA input during library preparation also affects the microbial organism representation and the gene expression pattern observed in metatranscriptomics [26]. A kit that can handle low-input RNA has been found to be more efficient in microbial transcriptomics, such as the SMARTer Stranded RNA-Seq kit (Takara Bio USA, Inc., San Jose, CA, USA) [26]. The produced library can be sequenced in a next-generation sequencing platform. Currently, short-read sequencing is the most common approach in the gut microbiome field. Long-read metatranscriptomics sequencing has also been developed for soil microbiome [27] that may be readily transferred to gut microbiome research.

Before the application of high-throughput sequencing, microarrays were initially developed for profiling global DNA or RNA expression. Different from the sequencing-based strategy, microarrays provide a targeted approach for analysing microbial communities using pre-defined probes, as exemplified by the development of phylogenetic microarrays [12,28,29].

### 3.3. Bioinformatics

Given the complexity of the microbiome, metatranscriptomics sequencing usually yields a large dataset containing millions of microbial mRNA molecules. Several bioinformatics pipelines have been developed to process raw sequencing reads, such as SAMSA2 [30], MetaTrans [31], MetaPro [32], HUMAnN2 [33], MOSCA [34], IMP [35], FMAP [36], and the Human Small Intestine Microbiota Metatranscriptome Pipeline [37]. A detailed comparison of some of the pipelines can be found here [38]. Overall, the processing of raw reads usually includes quality control (e.g., FastQC [39], available at http://www.bioinformatics.babraham.ac.uk/projects/fastqc, accessed on 5 March 2025), quality filtering (e.g., Trimmomatic [40]), filtering of rRNA sequences (e.g., SortMeRNA [41]), and mapping to a functional reference database (e.g., bowtie2 [42], BWA [43], DIAMOND [44]). Preprocessed, high-quality reads can be assembled into putative transcripts using de novo assemblers, such as the metagenomics assembler MEGAHIT [45] and the metatranscriptomics assembler IDBA-MT [46]. Assemblers affect the quality of sequence mapping. Given the unique features of transcripts in microbiomes, metatranscriptomics assemblers such as IDBA-MT may offer better resolution than metagenomic assemblers [38].

Taxonomy annotation can be performed with Kraken2 [47], MetaPhlan2 [48], and Kaiju [49] to identify microbial taxa. In addition to taxonomical and gene annotation, it is of common interest to determine what functions are differentially expressed between communities. Differential gene expression analysis can then be performed using statistical algorithms such as EdgeR [50] and DeSeq2 [51] in the R package. Readers are encouraged to refer to the specific package documentation for detailed instructions. With the diverse tools available, bioinformatics pipelines can be tailored depending on the experimental purpose. One of the challenges is that intense computing resources need to be secured before starting a metatranscriptomics pipeline.

## 4. Utilization of Metatranscriptomics in Food Science

### 4.1. Integrating Metatranscriptomics with Multi-Omics to Understand Food Fermentation

Fermented foods have been part of the human diet for a long time. They are considered to benefit gut health by regulating the intestinal digestibility of complex carbohydrates and proteins, affecting the production of bioactive compounds, and modulating the gut microbiome [52]. The quality and flavour of these foods largely depend on the microbial community and its fermentation activities [53]. Given the diverse microbial communities involved, a high-coverage approach to microbial community analysis is necessary.

**Figure 2:**
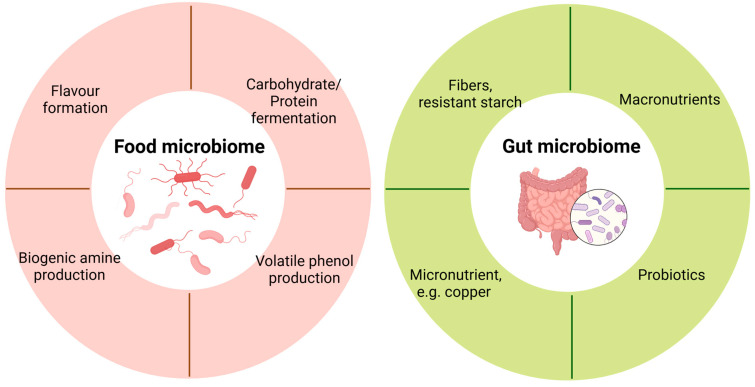
Application of metatranscriptomics in food and gut microbiome.

Metatranscriptomics sequencing has been employed to understand microbial processes involved in flavour formation during food fermentation (Table 1, Figure 2), such as the fermentation of liquor [54], sauce [55,56], vegetables [57,58,59,60,61,62,63], and fruit [64]. A diverse population of microbes has been identified to contribute to flavour formation. For instance, during the fermentation of noni fruit (*Morinda citrifolia* L.), *Acetobacter* sp. and *Acetobacter aceti* are the dominant species, while *Gluconobacter* sp. increases during the later phase of fermentation. This microbial shift correlates with decreases in pH, flavonoids, and total phenols, as well as an increase in titratable acid and microbial enzymes such as glycosyl transferases and carbohydrate-binding modules, as revealed by metatranscriptomics sequencing [64]. This observational study indicates a microbiome-enzyme-metabolite axis that contributes to flavour formation during food fermentation.

Metabolomics is a comprehensive analytical approach that measures small molecule metabolites in biological systems, providing important functional insights into biochemical processes and metabolic pathways within microorganisms. When combined with metabolomics, metatranscriptomics can provide a detailed view of microorganism-metabolite relationships based on the inferred functional relevance. Such analyses have been applied to various fermentation processes, including fermented vegetables [59,61,62,63] and liquor [54]. As reported in the fermentation of bamboo shoots, a time-series metatranscriptomics and metabolomics analysis identified *Lactococcus*, *Enterococcus*, *Leuconostoc*, *Lactiplantibacillus*, and *Weissella* as the major genera contributing to lactic, acetic, malic and citric acid production during the first six days. Subsequently, *Lactiplantibacillus* became predominant in producing these organic acids [61]. The shift is parallelled by changes in enzymatic function attributed to different microbial taxa. For example, pyruvate oxidase (EC: 1.2.3.3), which is involved in acetate synthesis, was primarily annotated to *Lactococcus* during the first six days but shifted to *Lactiplantibacillus* in the later phase [61]. Beyond carbohydrate metabolism, the microbiome also plays a significant role in amino acid metabolism. For instance, during chilli pepper fermentation, inoculation with *Staphylococcus succinus* increased the production of flavour compounds by up-regulating microbial enzymes involved in aroma compound synthesis and amino acid metabolism [59]. Overall, the combination of metabolomics and metatranscriptomics provides a powerful tool for unravelling the complex interactions between microorganisms and metabolites, providing a methodology to study metabolic dynamics in food fermentation.

Understanding the complex microbial community at the species level can help dissect the function of specific microbes. Species-level mapping could be achieved by metatranscriptomics or metagenomics sequencing and genome assembly. Species will be assigned based on reference genomes. However, it may be limited by distinguishing between highly similar strains. Different strains of the same species may differ in functional capabilities. Strain-level profiling could be conducted using single-nucleotide variants (SNVs) and genome reconstruction or culture-based approaches. In combination with curated genome annotation information, metatranscriptomics can provide species-level resolution mapping to unique microbial genomes, as demonstrated with *Lactobacillus sakei* [76] and *Weissella koreensis* [77]. In kimchi fermentation, the transcriptional activity of *Lactobacillus sakei* undergoes dynamic changes, with carbohydrate metabolism and the heterolactic fermentation pathway increasing in the later phase [76]. Genome mapping further reveals that L-lactate dehydrogenase, rather than D-lactate dehydrogenase, is the predominant enzyme responsible for L-lactate production in this species [76]. Similarly, genes associated with the heterolactic fermentation pathway of *Weissella koreensis* are upregulated during the later phases of fermentation [77], suggesting a coordinated response during fermentation.

These insights gained from species-level analysis highlight the importance of targeting specific functional microbes. By identifying key metabolic pathways and regulatory mechanisms in individual species, it is possible to develop strategies to selectively enhance or inhibit the activity of beneficial microbes during food fermentation.

### 4.2. Metatranscriptomics-Guided Enzyme Discovery During Food Fermentation

Since flavour formation is mediated by microbial enzymes, the identification, and characterization of key enzymes are critical steps for improving fermentation efficiency. Metatranscriptomics offers a significant advantage in this sense, as it can reveal the expression profiles of microbial genes involved in enzymatic processes. This provides valuable insights into which enzymes are active during fermentation and how they contribute to the production of flavour compounds.

One notable example is the fermentation of vegetables, where *Debaryomyces hansenii* and *Lactobacillus vermolensis* have been identified as prominent producers of the flavour compound vinylphenols [60]. The formation of these compounds is catalysed by a specific enzyme, phenolic acid decarboxylase, which is present in *Lactobacillus vermolensis* and elevated, as shown by metatranscriptomics. This enzyme facilitates the conversion of phenolic acids into aromatic compounds such as 4-vinylphenol and 4-vinylguaiacol, which are crucial for the development of distinctive flavours [60]. Interestingly, the inclusion of purified phenolic acid decarboxylase has been shown to accelerate fermentation processes, highlighting the enzyme’s potential for enhancing flavour production [78]. Of great relevance is the potential application of active enzymes in large-scale fermentation, which involves the mass production of enzymes using molecular cloning and genetic modification to enhance efficiency and stability. The theory of enzyme engineering can be further employed to optimize kinetic parameters with improved enzyme activity. This underscores the importance of integrating metatranscriptomics with enzymology to identify key microbial enzymes during fermentation processes.

### 4.3. Inter-Kingdom Impact Revealed by Metatranscriptomics

**Figure 3:**
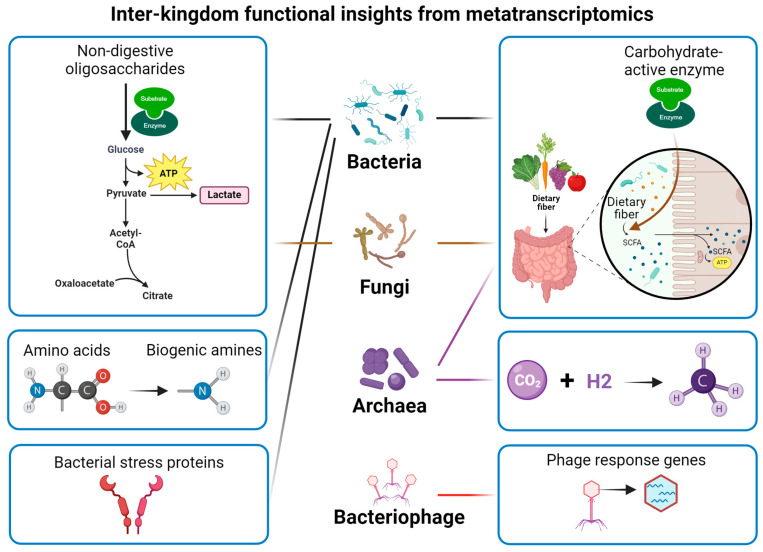
Functional insights into microbiome from metatranscriptomics.

Much of the current studies usually focus on the alteration of bacterial taxa. It should be noted that fungal species, such as *Saccharomyces cerevisiae*, *Yarrowia lipolytica*, and *Komagataella phaffii*, have also been used for food fermentation [79]. The shared fermented substrate leads to complex interactions between bacteria and fungi during fermentation, collectively affecting flavour formation [80]. Additionally, viruses and bacteriophages also exist in the fermentation system [81,82], which may create an ecological environment with complex interactions and affect the quality of fermented food. Metatranscriptomics offers a high-coverage approach to uncover the functional complexity of microbial communities (Figure 3).

Food fermentation involves a dynamic alteration of microorganisms and flavour compounds. For example, during a 197-day fermentation of industrial Sichuan radish paocai, fungi such as *Debaryomyces* were actively contributing to flavour metabolism, including the production of acetic acid, other fatty acids, and sulfides in the first 100 days, while *Lactobacillus* dominated in the later phase, contributing to the production of acetic acid and lactic acid [58]. The shift in active microbes is parallel with an increase in enzymes related to the TCA cycle, propanoic acid metabolism, oxalic acid metabolism, and formic acid metabolism at first 100 days and then a decrease in these enzymes. Similar trends are observed in the solid-state fermentation of soy-sauce-aroma-type liquor, where fungi species like *Pichia* and *Schizosaccharomyces* are responsible for ethanol production via aldehyde dehydrogenase in the early phase, while *Lactobacillus* species are predominant in producing lactic acid via lactate dehydrogenase later [68]. The findings clearly define the different roles of fungi and bacteria in affecting the fermentation processes.

Cheese ripening is a complex biochemical process that involves significant alterations in the metabolic activities of bacteria and fungi. Key microbial species such as *Brevibacterium aurantiacum*, *Streptococcus thermophilus*, *Lactobacillus delbrueckii* ssp. *bulgaricus*, *Debaryomyces hansenii*, and *Geotrichum candidum* play critical roles in these transformations. Among these, yeast species like *D. hansenii* and *G. candidum* are particularly active members, contributing to the upregulated genes of amino acid catabolism, which enhances flavour development, and the downregulated genes of galactose catabolism and bacterial energy production [66]. These microbial activities lead to the formation of key volatile compounds and textures that characterize the unique qualities of ripened cheese. With the important role of fungi in regulating the fermentation process, a future study may explore the combination of active bacteria and fungi to modulate cheese flavour.

In addition to bacteria and fungi, other less dominant microorganisms, such as viruses and phages, are non-dominant within the microbiome community but may play significant roles in shaping microbial ecosystems. Beyond serving as a regulator of the bacterial population, the phages further regulate microbial metabolism. As proved in a gnotobiotic mouse model, phage colonization leads to a wide impact on the gut metabolome, including 860 metabolites involved in amino acids, carbohydrates, lipids, and cofactor/vitamin metabolism [83]. These findings highlight the metabolic role of phages beyond the general ecological significance. Detection of viruses and phages in fermented food, such as cheese, cucumber, soybean, and kimchi, has been reported [82]. However, only a few studies examine the transcriptional activity of these unique microbial populations. For example, metatranscriptomics profiling in kimchi fermentation revealed a high abundance of pepper mild mottle virus, followed by garlic virus A. Interestingly, the transcriptional activity of the plant virus RNA decreased from day 7 to day 29 of fermentation [84]. The reason leading to this dynamic behaviour remains unknown but may be due to the altered environment and substrate availability resulting from the co-existing bacterial and fungal activity. This highlights the need for further research into how non-dominant microorganisms, including viruses and phages, contribute to the overall dynamics of microbial communities in fermented foods.

### 4.4. Fibre Degradation in Food Fermentation

The ability of microorganisms to produce carbohydrate-active enzymes (CAZymes) positions them as key players in the breakdown and utilization of food fibres, which in turn facilitates the fermentation process. As previously noted, metatranscriptomics provides an ideal tool for investigating microbial activities, particularly their response to fibres in fermentation environments. This method enables the capture of active transcriptions of genes responsible for CAZyme production, shedding light on the metabolic strategies employed by microbes at the enzyme level.

An example is cocoa fermentation, where a consortium of microorganisms plays a pivotal role in transforming key substrates like glucose, fructose, and citric acid into fermentation products such as lactic acid, mannitol, and acetic acid. Microbes like *Limosilactobacillus fermentum*, *Liquorilactobacillus cacaonum*, *Acetobacter pasteurianus*, *Hanseniaspora opuntiae*, and *Saccharomyces cerevisiae* are actively involved in these transformations, driving the fermentation process and contributing to the development of flavours and aromas in the final product [69]. Of particular interest is that the fibrous pectin component in coca pulp that contains complex carbohydrates can also be degraded during fermentation through the glycoside hydrolase families (GH2, GH43) produced by *Paucilactobacillus vaccinostercus* or polysaccharide lyase families (PL4, PL9) produced by *Pectobacterium* spp. [69]. Thus, it is clear that different microorganisms undertake distinct functional roles in effectively fermenting foods. The ability of different microbial species to contribute specific enzymatic functions suggests the functional complexity of the microbiome ecosystem.

### 4.5. Bioconversion of Food Ingredients

Another application of metatranscriptomics is to understand the bioconversion mechanisms of dietary ingredients by the gut microbiome. Dietary compounds such as isoflavones and polyphenols in soy products have multiple health benefits, including regulating host immunity, oxidative stress, and gut microbiome [85]. A notable microbial metabolite, equol, derived from soy isoflavones, exhibits various physiological effects. Gut microbes are involved in metabolizing soy isoflavones into equol. As shown in mice fed with synbiotics comprising lactulose and *Lactobacillus rhamnosus* ATCC 7469, the bioconversion of soy isoflavones to equol was enhanced, an effect due to the upregulation of microbial pyruvate kinase in the glycolysis/gluconeogenesis pathway [86].

Biogenic amines are byproducts of microbial decarboxylation during the fermentation of protein and amino acids. Metatranscriptomic profiling during the fermentation of ganjang, a traditional Korean soy sauce, identified several microbes actively producing biogenic amines [56]. For instance, *Tetragenococcus* and *Virgibacillus* were major genera producing tyramine via tyrosine decarboxylase, which is underrepresented in metagenomics studies. *Lactobacillus* and *Halomonas* were the major genera producing putrescine via ornithine decarboxylase. *Staphylococcus* was the primary genus for cadaverine production via lysine decarboxylase [56]. This taxa-specific metabolism highlights the distinct functional roles of different taxa in producing biogenic amines. Understanding microbial decarboxylation functions can help develop interventions to reduce the accumulation of biogenic amines during fermentation.

## 5. Utilization of Metatranscriptomics in Nutrition Science

### 5.1. Interaction Between Gut Microbiome and Fibres Revealed by Metatranscriptomics

As a fundamental food ingredient in the diet, dietary fibre exerts multiple benefits, including regulating intestinal transit and the gut microbiome and promoting the production of short-chain fatty acids [87]. It has also been used to regulate gastrointestinal disorders. Dietary fibre is primarily utilized by gut microbiota in the large intestine via carbohydrate-active enzymes. For example, a recent study uncovered a novel role of the human gut microbiome in utilizing crystalline cellulose through functional multienzymatic cellulosome systems [88]. The intestinal fermentation by the gut microbiome leads to the production of byproducts such as hydrogen and formate. Accumulation of these products will increase the H2 partial pressure and reduce fermentation efficiency [89]. In brief, fibre degradation involves the activity of multiple microorganisms and different enzymes. Metatranscriptomics provides a powerful tool to map the intestinal microbes that execute specific functionalities in response to dietary fibres (Figure 2).

Isomalto/malto-polysaccharides, a type of dietary fibre, are primarily degraded by *Bacteroides*, *Lactobacillus*, and *Bifidobacterium* in the human colon through carbohydrate-active enzymes and microbial enzymes involved in short-chain fatty acid production [90]. Studies in rats consuming resistant starch have shown an increase in active *Prevotella*, *Subdoligranulum*, *Bacteroides*, and *Parabacteroides*, while *Desulfovibrio* and *Rosiburia* decrease in the cecum [91]. *Bacteroides* and *Parabacteroides* are major genera producing starch-degrading enzymes, including 4-α-glucanotransferase and glycosidases [91], while *Bacteroides* species exhibit broad activities in degrading complex oligosaccharides. At the functional level, resistant starch also upregulates microbial genes related to molecular chaperone, starch degradation, and carbohydrate metabolism [91]. These observations highlight the varied roles of microbes in utilizing different substrates.

Similar results are observed in human trials. Increasing dietary fibres from 10 to 40 g per day promotes active *Bacteroides* and *Bifidobacterium* in the human gut, along with the upregulation of amylase and downregulation of microbial enzymes related to mucin degradation such as endo-a-N acetylgalactosaminidases, α-glucuronidases, and α-L-arabinofuranosidases [92]. Of particular interest is the upregulation of methanogenesis in archaeal species by dietary fibres.

Methanogenic archaea are present in low abundance in the gut. Nevertheless, they have a fundamental role in the intestinal fermentation. Gut bacteria could utilize complex carbohydrates to produce simple sugar and organic acids, as well as gases such as hydrogen. An accumulation of hydrogen will slow down the fermentation process. These methanogenic archaea, or methanogens, are indispensable in removing H2 from bacterial fermentation and reducing H2 partial pressure, thereby maintaining the fermentation process [93]. Upregulation of methanogenesis after a high-fibre diet has been observed in humans [92] and animal models [94,95]. Key biomarkers from metatranscriptomics include enzymes for methanogenesis, such as methyl-CoM reductase, which is upregulated by dietary fibre [92]. Additionally, dietary fibres reduce oxidative and stress-related activities in the gut microbiome [92,94] and upregulate phage response genes [92], indicating diverse regulations on gut health.

Maintaining oxygen balance is fundamental for gut homeostasis, particularly in the large intestine, where it is inhabited by strictly anaerobic microorganisms. An increase in oxygen availability in the large intestine can disturb the microbiome and redox homeostasis [72,96]. This disturbance has been observed in the gut of antibiotic-treated mice [95], but it could be reversed by dietary fibres. In antibiotic-treated mice supplemented with dietary fibre, an increase in carbohydrate-active enzymes involved in pectin and inulin utilization has been found [95]. Dietary fibre promotes a low-oxygen fermentative gut microenvironment by enhancing microbial processes, including carbon fixation, methanogenesis, and anabolic metabolism. It further restores antibiotic-induced dysregulation of the redox potential with a decrease in bacteria capable of producing Complex I enzyme, particularly those in Enterobacteriaceae, which collectively contributes to low redox potential after dietary fibre intake [95]. Another study in a pig model also supports the benefit of dietary fibre in reducing redox potential in the gut microbiome. Metatranscriptomics analysis of the colonic microbiome reveals that dietary pectin increases hydrogenotrophic taxa, such as *Desulfovibrio* and *Methanobrevibacter smithii*, while decreasing *Kazachstania*, a fungus with abundant modules involved in oxidative phosphorylation. Collectively, these alterations contribute to a low redox potential in the colon of pigs [94]. In summary, dietary fibres may favourably regulate the oxygen balance and redox homeostasis in the gut microbiome.

### 5.2. Metatranscriptomics Identify Biomarkers of Dietary Style, Macronutrients, and Micronutrient Intake

Accumulating studies have proved that diet is a major driver in shaping the gut microbiome [97,98]. Dietary carbohydrates, fats, and proteins are essential substrates for both the gut microbiome and the bodily system. The gut microbiome is equipped with enzymes to metabolize these substrates. Therefore, changes in dietary patterns and macronutrients could influence the gut microbial composition [99]. The compositional alteration is accompanied by changes in microbial functions. For instance, metatranscriptomics has unveiled that Erysipelotrichaceae species and their peptidase and amino acid metabolism enzymes are sensitive to an increase in dietary high protein [100], while *Anaerostipes* and microbial cellular stress and immune response genes are provoked in simple carbohydrates relative to refined carbohydrate in the diet [101]. Now, it is clear that the microbes are armed with different enzymes and transporter systems to utilize nutrients from the environment.

Dietary patterns affect the gut microbiome. Due to variations in dietary macronutrient composition, different dietary patterns could shape the microbial landscape greatly. As demonstrated in the human gut, vegetarians exhibit higher *Prevotella copri* and lower *Bacteroides fragilis*, *Bilophila wadsworthia*, and *Parabacteroides distasonis* than omnivores, which collectively leads to increased metabolism of branched-chain amino acids causing reduced branched-chain amino acids in the blood of vegetarians [102]. The high-carbohydrate nature of a vegetarian diet relative to an omnivore diet is also reflected in the increased butyrate production [102] originating from carbohydrate fermentation. Butyrate has multifunctional roles as a major energy source for colonocytes [103]. Notably, an increase in butyrate production has been reported in adults consuming peanuts when compared to those consuming low-fat, high-carbohydrate snacks [104]. Functionally, the increased butyrate is attributed to the enrichment of *Roseburia* bacteria and the enzyme aerobic carbon-monoxide dehydrogenase small subunit [104]. The response of *Roseburia* further validates its role as a major butyrate-producing bacteria in the human gut [105]. Known as a health-promoting metabolite, butyrate has wide physiological effects that offer an avenue to develop butyrate-based interventions to harness gut health.

Dietary micronutrients, including iron, zinc, copper, and calcium, have been linked to alterations in microbial compositions [106,107]. Despite the established influence of these minerals on microbial populations, their impact on gut microbial functions is less thoroughly understood. A recent study utilizing in vitro culture of faeces from children revealed that the inclusion of high copper ion (Cu^2+^) at 4 mg/L, relative to 2 mg/L, decreases *Lactobacillus* and *Lactococcus* abundances and decreases microbial activities involved in antioxidant and detoxification, as well as decreases in several metabolism processes including propionate, butyrate, pyruvate, and methanogenesis [108]. These findings suggest the importance of mineral balance in regulating gut health. While the nutritional significance of micronutrients has been widely appreciated, their impact on the gut microbiome needs more investigation, particularly the microbial functions and host-microbiome interaction.

### 5.3. Metatranscriptomics in Understanding Probiotic Action in the Gut

Probiotics are live microorganisms that, when administered in adequate amounts, confer a health benefit on the host [109]. Probiotics have been found to affect the function of multiple organs, including the gut, liver, and brain [110,111,112,113]. In the gut, probiotics are widely recognized to exert benefits in regulating the gut microbiome structure, immune response, and metabolism [114,115]. It is noteworthy that the benefits of probiotics on gut health are maximal at the active status, a character well within the scope of metatranscriptomics that captures microbial activities.

By mapping the sequencing read from a mixture community to those of probiotic strains, it is possible to analyse the functional dynamics of probiotics. By monitoring the transcriptome of *Lacticaseibacillus casei Zhang* in vivo and comparing with in vitro transcriptome profiles, Wang and colleagues show that around 39% of mRNAs were inhibited in vivo, and the expression varies at different time points. For instance, microbial genes involved in the amino acid metabolism of *L. casei Zhang* were induced at day 14 of ingestion, while those involved in galactose, propanoate, and butyrate metabolism were induced at day 28 of ingestion [116], indicating a time-dependent manner of probiotic activity.

The strategy has also proven successful in understanding the probiotic function in the human small intestine. During 12 h consumption of a dairy product fermented by *Lacticaseibacillus rhamnosus* CNCM I-3690, a series of transcriptional alterations occur in the small intestine, including the upregulation of microbial genes encoding carbohydrate nutrient acquisition (e.g., sucrose, mannose, sorbitol, and ascorbate), ribosomal translational proteins, and surface proteins (e.g., fibronectin-binding and peptidoglycan-binding proteins) for adherence function in *L. rhamnosus* compared to gene expressions in vitro [117]. Expressions of enzymes related to inositol degradation and glutamate synthesis II are also regulated in the small intestine [117]. However, the probiotic administration has minor effects on the other indigenous microbiome. It is interesting to note that the pathways involved in galactose metabolism, ascorbate, and aldarate metabolism are all induced after administration with *L. casei Zhang* [116] and *L. rhamnosus* CNCM I-3690 [117]. This probably represents a consistent regulation mechanism by *Lacticaseibacillus* probiotics.

## 6. Perspectives and Conclusions

Advances in microbiome science and next-generation sequencing techniques have significantly enriched our knowledge regarding the structural and functional diversity in both food and gut microbiomes. Metatranscriptomics offers a unique approach to comprehensively understanding the active microorganisms and functional modules, especially those involved in dynamic microbial processes such as flavour formation, carbohydrate-active enzymes, and the survival of probiotics (Figure 3). However, it should be noted that the correlation of transcripts to metabolites does not establish causality when combining metatranscriptomics with metabolomics. The mechanism of action may be further understood by implementing engineering microbes and enzymes.

The gut microbiome is complex in both composition and function, giving rise to the extensive interaction with the intestinal epithelium and extraintestinal organs. The capability to capture the transcriptional activity of bacteria, fungi, archaea, and bacteriophages further broadens the application in inter-kingdom research. Nevertheless, the application of metatranscriptomics may be limited due to the high costs and technical barriers, such as intensive computing requirements, limitations in detecting non-dominant microbial transcripts, and challenges in transcript annotation for non-bacterial communities, including fungi and archaea. Other limitations need to be considered as well, such as quantitative bias, the function of “hypothetical” or poorly characterized proteins, the correlation of the metatranscriptome to viability and activity, extrapolation to distal sites of the intestine using faecal samples, and difficulties in separating prokaryotic mRNA from rRNA.

While metatranscriptomics provides valuable insights into microbial functionalities, it is important to note that the integration of metatranscriptomics with metagenomics and metabolomics could collectively offer a holistic view of the microbiome. As discussed in the research on the fermented food microbiome, combining metatranscriptomics with metabolomics has enabled the generation of an integrative metabolic network that can be manipulated to affect flavour formation. Moreover, identifying enzymes responsible for metabolizing carbohydrates, amino acids, or fibres will offer unique insights into the development of enzymes for targeted control of food fermentation or gut-based manipulation for promoting gut homeostasis.

## Figures and Tables

**Table 1:** Summary of metatranscriptomics studies in food fermentation.

Food	Research Purpose	Approach	Functional Microorganisms	Major Functions	Reference
Fermented Bamboo shoot	Flavour formation	Metatranscriptomics Metabolomics	*Lactococcus, Enterococcus, Leuconostoc, Lactiplantibacillus* and *Weissella*	Lactic, acetic, malic and citric acid synthesis	[61]
Fermented Laotan Suancai	Flavour formation	Metatranscriptomics Metabolomics	*Companilactobacillus alimentarius, Weissella cibaria, Lactiplantibacillus plantarum,* and *Loigolactobacillus coryniformis*	Oxalic acid and lactic acid synthesis, Geranyl-PP metabolism	[63]
Fermented Laotan Suancai	Biogenic amine production	Metatranscriptomics Metabolomics	*Lactobacillaceae Species* and *Tetragenococcus halophilus*	Putrescine and histamine synthesis	[62]
Fermented Kimchi	Lactic acid bacteria succession	Metagenomic Metatranscriptomics	*Lactobacillus graminis, Lactobacillus curvatus, Lactobacillus sakei sub*sp. *carnosus, Weissella viridescens*	Lactic acid synthesis	[57]
Fermented Unpolished Black Rice	Production of Melanogenesis Inhibitors	Metatranscriptomics	*Saccharomyces cerevisiae, Saccharomycopsis fibuligera, Rhizopus oryzae,* and *Pediococcus pentosaceus*	Species interaction leads to maximum melanogenesis inhibition activity	[65]
Reblochon-Style Cheese	Microbial activity during cheese ripening	Metatranscriptomics	*Debaryomyces hansenii,* *Geotrichum candidum*	Amino acid catabolism	[66]
Swiss-type Maasdam cheese	Microbial activity during cheese ripening	Metagenomic Metatranscriptomics	*Lactococcus lactis*	Upregulated vitamin biosynthesis and homolactic fermentation during cold room ripening	[67]
Soy sauce aroma type liquor	Flavour formation	Metatranscriptomics Amplicon sequencing	Stage 1: *Schizosaccharomyces* Stage 2: *Lactobacillus*	Stage 1: Ethanol production Stage 2: Lactic acid and acetic acid production	[68]
Fermented Costa Rican Cocoa Box	Microbial composition and activity	Metagenomics Metatranscriptomics	*Limosilactobacillus fermentum,* *Liquorilactobacillus cacaonum, Lactiplantibacillus plantarum, Acetobacter pasteurianus, Acetobacter ghanensis, Hanseniaspora opuntiae* and *Saccharomyces cerevisiae*	Utilization of glucose, fructose, and citric acid to produce ethanol, lactic acid, acetic acid, and mannitol	[69]
Fermented ganjang (Korean traditional soy sauce)	Biogenic amine production	Metagenomics Metatranscriptomics	*Staphylococcus produces cadaverine; Tetragenococcus produces histamine; Lactobacillus* and *Halomonas produce putrescine; Tetragenococcus, Bacillus, and Enterococcus produce tyramine*	Biogenic amine synthesis	[56]
Fermented dajiang (soybean Paste)	Flavour formation	Metatranscriptomics	*Lactobacillus produces acetic acid and ethanol; Tetragenococcus produces aldehydes* and *ketones*	Flavour metabolite synthesis	[70]
Fermented Sichuan radish paocai	Flavour formation	Metatranscriptomic	*Lactobacillus, Debaryomyces*	flavour metabolism, such as acetic acid and lactic acid	[58]
Fermented Sichuan radish paocai	Aromatic volatile phenol production	Metatranscriptomic	*Lactobacillus vermolensis*	Produce phenolic acid decarboxylase for the decarboxylation of p-coumaric acid, ferulic acid, and caffeic acid into 4-vinylphenol and 4-vinylguaiacol	[60]
Fermentation of sour juice and drying of milk fan	Flavour formation	Metatranscriptomic	*Lactococcus, Rhodotorula, Candida, Cutaneotrichosporon, Yarrowia*	Positive association with aroma-active compounds, including ethyl acetate, 2-heptanone, isovaleraldehyde, butyric acid, nonanal, and hexanal.	[71]
Fermented noni fruit	Odour formation	Metatranscriptomic	*Acetobacter* sp., *Acetobacter aceti*, and *Gluconobacter* sp.	Carbohydrate metabolism, acetic acid production	[64]
Kimchi	Ultra-small microbiome	Metatranscriptome Metataxonome	*Lactobacillus, Leuconostoc, Weissella, Akkermansia*	Lactic acid production, protein metabolism	[72]
Niulanshan Baijiu Fermentation	Flavour formation	Metatranscriptomics Metabolomics	*Streptococcus, Lactobacillus,* *Pediococcus, Campylobacter, Yersinia, Weissella, Talaromyces, Aspergillus, Mixia, Rhizophagus,* and *Gloeophyllum*	Produce volatile compounds, such as 3-methylbutanol, 2-methylpropanoate, 3-methylbutal	[54]
Fermented chilli pepper	Flavour formation	Metatranscriptomics Metabolomics	*Staphylococcus, Lactobacillus*	Esters, terpenes, alcohols, aspartic acid and glutamic acid production	[59]
Sugarcane vinasse	Vinasse metabolism	Metatranscriptomics	*Pectinatus, Megasphaera, Clostridium, Pectinatus frisingensis*	Acetate or propionate production	[73]
Fermented soybean	Microbial community	Metagenomic Metatranscriptomics	*Providencia stuartii*	Carbohydrates, protein, energy, and amino acid metabolism	[74]
Shrimp sauce	Flavour formation	Metatranscriptomics	*Tetragenococcus halophilus*	Citrate cycle and oxidative phosphorylation	[55]
Traditional Italian Caciocavallo Silano cheese	Cheese ripening	Metatranscriptomics	*Lactobacillus casei, Lactobacillus buchneri*	Proteolysis, lipolysis, and amino acid/lipid catabolism	[75]

## Data Availability

This study did not generate new data.
